# Screening for TORCH Antibodies in Croatian Childbearing-Aged Women, 2014–2023

**DOI:** 10.3390/antib13020049

**Published:** 2024-06-18

**Authors:** Tatjana Vilibic-Cavlek, Branko Kolaric, Marko Belamaric, Mario Sviben, Thomas Ferenc, Dan Navolan, Viktor Bekic, Ljiljana Milasincic, Ljiljana Antolasic, Maja Vilibic, Mateja Vujica Ferenc, Ema Reicher, Tadej Jezek, Ioana Ciohat, Raluca Catalina Parvanescu, Matea Kos, Maja Bogdanic

**Affiliations:** 1 Department of Virology, Croatian Institute of Public Health, 10000 Zagreb, Croatia; viktor.bekic@hzjz.hr (V.B.); ljiljana.milasincic@hzjz.hr (L.M.); ljiljana.antolasic@hzjz.hr (L.A.); maja.bogdanic@hzjz.hr (M.B.); 2 School of Medicine, University of Zagreb, 10000 Zagreb, Croatia; mario.sviben@hzjz.hr (M.S.); ema.reicher@gmail.com (E.R.); tadej123jezek@gmail.com (T.J.); 3 Andrija Stampar Teaching Institute of Public Health, 10000 Zagreb, Croatia; 4 Department of Social Medicine and Epidemiology, Medical Faculty, University of Rijeka, 51000 Rijeka, Croatia; 5 Teaching Institute for Emergency Medicine, 10000 Zagreb, Croatia; mbelamaric123@gmail.com; 6 Department of Parasitology, Croatian Institute of Public Health, 10000 Zagreb, Croatia; 7 Department of Diagnostic and Interventional Radiology, University Hospital Merkur, 10000 Zagreb, Croatia; thomas.ferenc95@gmail.com; 8 Department of Obstetrics and Gynecology, ‘Victor Babes’ University of Medicine and Pharmacy, 300041 Timisoara, Romania; navolan@umft.ro; 9 Department of Psychiatry, Sestre Milosrdnice University Hospital Center, 10000 Zagreb, Croatia; maja.vilibic@gmail.com; 10 School of Medicine, Catholic University of Croatia, 10000 Zagreb, Croatia; 11 Department of Obstetrics and Gynecology, University Hospital Centre Zagreb, 10000 Zagreb, Croatia; matejavujica1@gmail.com; 12 Antenatal Medicine Laboratory, Timisoara City Emergency Hospital, 300202 Timisoara, Romania; tucaioana@yahoo.com (I.C.); dr.ralucaparvanescu@gmail.com (R.C.P.); 13 Synlab Polyclinic for Medicine Laboratory Diagnostics, 10000 Zagreb, Croatia; kos.matea90@gmail.com

**Keywords:** TORCH, *Toxoplasma gondii*, rubella virus, cytomegalovirus, herpes simplex viruses, childbearing-aged women, Croatia

## Abstract

TORCH infections usually result in mild maternal morbidity, but may cause severe congenital abnormalities. Therefore, it is important to detect maternal infections, monitor the fetus after the disease has been recognized, and define the seronegative women who are at risk of primary infection during pregnancy. From 2014 to 2023, serum samples from 1032 childbearing-aged and pregnant women (16–45 years) were tested for IgM/IgG antibodies to the most common TORCH pathogens: *Toxoplasma gondii*, rubella virus (RUBV), cytomegalovirus (CMV), and herpes simplex viruses (HSV-1 and HSV-2). The overall IgG seroprevalence rates were 20.1% for *T. gondii*, 91.3% for RUBV, 70.5% for CMV, 66.8% for HSV-1, and 3.5% for HSV-2. Only HSV-2 seroprevalence was age-related, with a significant progressive increase in seropositivity from 0% in those aged less than 26 years to 9.3% in those older than 40 years. The seroprevalence of *T. gondii* was higher in residents of suburban/rural areas than in residents of urban areas (27.4% vs. 17.1%). In addition, participants from continental regions were more often toxoplasma-seropositive than those from coastal regions (22.2% vs. 15.3%). HSV-1 seroprevalence was also higher in suburban/rural areas (71.7% vs. 64.7%). Obstetric history was not associated with TORCH seropositivity. Univariate and multivariate risk analysis showed that suburban/rural areas of residence and continental geographic regions were significant risk factors for *T. gondii* seroprevalence. Furthermore, suburban/rural area of residence was a significant risk factor for HSV-1 seroprevalence, while older age was a significant risk factor for HSV-2 seroprevalence. A declining trend in the seroprevalence of all TORCH pathogens was observed compared to previous Croatian studies (2005–2011). Similarly, the proportion of women simultaneously IgG-seropositive to two or three pathogens decreased over time. The maternal serology before pregnancy could potentially reduce the burden of congenital TORCH infections.

## 1. Introduction

TORCH—*Toxoplasma gondii*, others (*Treponema pallidum*, varicella-zoster virus, parvovirus B19, etc.), rubella virus (RUBV), cytomegalovirus (CMV), and herpes simplex viruses type 1 and 2 (HSV-1, HSV-2)—encompasses some of the most prevalent pathogens associated with congenital abnormalities. The majority of TORCH infections result in mild maternal morbidity, but have severe fetal consequences, and treatment of the mother’s illness often does not affect the fetal outcome. Therefore, it is important for clinicians to identify maternal infections and monitor the fetus once the disease has been identified [1]. In addition, it is important to define the seronegative women who are at risk of primary infection during pregnancy [2].

Toxoplasmosis is a parasitic infection caused by the protozoon *T. gondii*. The majority of immunocompetent individuals do not develop symptoms or might have nonspecific flu-like symptoms and lymphadenopathy [3]. Congenital toxoplasmosis is a complication of a primary maternal *T. gondii* infection during pregnancy. The mother’s immune system, the virulence of the strain, the parasite load, and the gestational age at which the mother was infected all affect the severity of a newborn or fetal disease [4]. The risk of *T. gondii* transmission increases with increasing gestational age, but the disease severity decreases [5]. The spectrum of clinical manifestations of congenital toxoplasmosis varies from mild symptoms to severe consequences, such as chorioretinitis, hydrocephalus, microcephaly, mental retardation, and even death [6].

Rubella is a viral disease caused by RUBV. It is a highly contagious but generally mild and in most cases self-limiting disease. However, maternal RUBV infection during the first trimester of pregnancy can cause congenital rubella syndrome (CRS). CRS represents a global public health concern, with more than 100,000 estimated annual reported cases worldwide. The risk of transplacental transmission depends on the time of infection. If a maternal infection occurs within the first 12 weeks of gestation, up to 85% of newborns will have congenital defects compared to 50% in maternal infections within 13 to 16 weeks of gestation and 25% in maternal infections during the second part of the second trimester [7]. Congenital heart diseases, cataracts, hearing impairment, and developmental delay are common congenital abnormalities associated with CRS [8].

CMV is a widely distributed human beta-herpesvirus. CMV commonly causes asymptomatic or mild mononucleosis-like disease in immunocompetent children and adults; however, congenital CMV infection is a public health problem, affecting 0.67% of live births [9,10]. Congenital infection may occur in primary or recurrent CMV infection (reactivation or reinfection with a different viral strain). Although mostly asymptomatic, primary maternal CMV infection in pregnancy poses the highest risk of transplacental transmission (30–35%) compared to 1.1–1.7% for non-primary infections. Fetal abnormalities include intrauterine growth restriction, intracranial calcifications, microcephaly, ventriculomegaly, chorioretinitis, and hepatomegaly [11].

HSV-1 and HSV-2 are among the most widely distributed viruses worldwide. HSV-1 predominates in orofacial lesions, while HSV-2 mainly causes genital herpes. Nevertheless, both these viruses can infect orofacial areas and the genital tract. For HSV infections that occur in the last trimester of pregnancy, the risk of neonatal infection ranges from 30% to 50%, while the risk for early pregnancy infections is only 1% [12]. Several forms of neonatal infections can be identified based on the time of maternal infection: intrauterine infections (5% of cases), postnatal infections (10% of cases), and perinatal infections (85% of cases) [13]. Spontaneous abortion, intrauterine growth retardation, preterm birth, and congenital and neonatal HSV infections are all linked to genital herpes infections during pregnancy. HSV infections in neonates infected intrapartum or postnatally can manifest as disease localized to the skin, eye, and/or mouth, HSV encephalitis, or disseminated HSV infection, with mortality rates of more than 80% in untreated patients [12].

Only a few studies have analyzed the prevalence of TORCH infections in Croatia. Toxoplasma seropositivity was analyzed in the childbearing-aged female population in Split-Dalmatia County (1994–1995), showing a seropositivity ranging from 28.1% to 42.6% [14]. A subsequent study conducted from 2005 to 2009 tested childbearing-aged women for *T. gondii*, RUBV, CMV, and HSV-1/2 [15]. Three other studies analyzed seroprevalence in pregnant women: HSV-1/2 (2008–2010 and 2011–2023) [2,16] and CMV (2013–2015) [17]. Since recent data are lacking, this study aimed to analyze the seroprevalence of and risk factors for TORCH infections in childbearing-aged women over a 10-year period (2014–2023).

## 2. Materials and Methods

### 2.1. Study Participants

**Figure 1:**
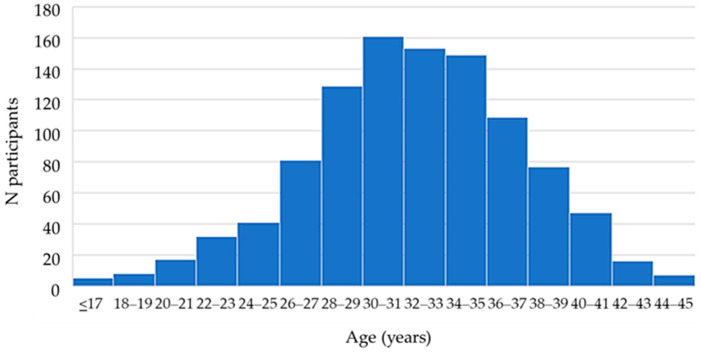
Distribution of study participants by age.

The study included 1032 childbearing-aged women (16–45 years; Figure 1) tested at the Croatian Institute of Public Health, the largest public health institute in the country. All women tested for TORCH pathogens consecutively from January 2014 to December 2023 were included. All of the participants were of Croatian nationality and there were no migrants tested. For this study, participants were classified according to age (a five-year age group), area of residence (urban or suburban/rural), geographic region (continental or coastal), and obstetric history (non-pregnant, normal pregnancy, unfavorable obstetric history: previous spontaneous abortions, children with congenital malformations, infertility).

### 2.2. Methods

Initial serological screening (IgM and IgG antibodies) was performed using commercial enzyme-linked immunoassay (ELISA) for RUBV (Novatec Immunodiagnostica, Dietzenbach, Germany), CMV (Vircell Microbiologists, Granada, Spain), and HSV-1/2 (Virotech Diagnostics, Dietzenbach, Germany) and enzyme-linked fluorescence assay (ELFA) for *T. gondii* (Vidas, Marcy-l’Étoile, France). IgM/IgG-positive samples were further tested for IgG avidity for *T. gondii* (Vidas, Marcy-l’Étoile, France), RUBV (Euroimmun, Lübeck, Germany), and CMV (Euroimmun, Lübeck, Germany), and Western blot (WB) was used for HSV-1/2 (Euroimmun, Lübeck, Germany) (Table 1).

### 2.3. Statistical Analysis

Differences between groups of categorical variables were assessed using chi-squared and Fisher’s exact tests. Odds ratios (ORs) and adjusted odds ratios (AORs) ± 95% confidence intervals (CIs) were used to assess the univariate and multivariate association of positive serological tests and explanatory variables (age, area of residence, geographic region, and obstetric history). Statistical analysis was performed using STATA/MP 17.0 for Windows (StataCorp LLC, Lakeway Drive, College Station, TX, USA). The level of statistical significance was set at *p* < 0.05.

## 3. Results

### 3.1. Characteristics of Study Participants

Mean participant age was 31.9 ± 5.1 years. The area of residence was urban for 725 (70.3%) of participants and suburban/rural for 307 (29.7%) of participants. The geographic region of residence was continental for 724 (70.2%) and coastal for 308 (29.8%) participants. Regarding obstetric history, 271 (26.3%) of women were not pregnant, 608 (58.9%) had a normal pregnancy and 153 (14.8%) reported an unfavorable obstetric history.

### 3.2. TORCH Seroprevalence

*Toxoplasma gondii* IgG antibodies were detected in 208 (20.2%; 95% CI = 17.8–22.7%) participants. *Toxoplasma gondii* IgM antibodies were found in four (0.4%; 95% CI = 0.1–1.0%) IgG-seropositive participants; however, all showed high IgG avidity, which ruled out acute toxoplasmosis. There was no significant difference in IgG seroprevalence according to age (18.8–25.6%). Significant differences were observed according to area of residence and geographic region. Participants from suburban/rural areas were more often seropositive compared to those from urban areas (27.4% vs. 17.1%; *p* < 0.001). In addition, higher seropositivity was observed in continental areas than in coastal areas (22.2% vs. 15.3%; *p* = 0.011). IgG seropositivity was not associated with obstetric history (non-pregnant women 23.6%, normal pregnancy 19.4%, unfavorable obstetric history 17.0%) (Table 2).

RUBV IgG antibodies were detected in 942 (91.3%; 95% CI = 89.6–93.0%) participants. RUBV IgM antibodies were found in two (0.2%; 95% CI = <0.1–0.7%) IgG-seropositive participants. Both participants showed high AI (77% and 83%, respectively). There was no significant difference in IgG seroprevalence according to age (81.3–95.4%), urban and suburban/rural area of residence (91.3% and 91.2%), continental and coastal geographic region (91.7% and 90.3%), or obstetric history (90.5–91.5%) (Table 3).

CMV IgG antibodies were detected in 728 (70.5%, 95% CI = 67.7–73.3%) participants. Sixty-five of the IgG-seropositive women were IgM-positive (8.9%, 95% CI = 6.9–11.2%); however, based on IgG avidity, only two showed low/borderline AI (35% and 51%, respectively), suggesting recent primary CMV infection. No significant differences in IgG seroprevalence were observed among age groups (62.5–72.4%), urban and suburban/rural areas of residence (69.1% vs. 73.9%), or continental and coastal regions (70.1% vs. 71.8%). Higher CMV IgG seropositivity was found in women with an unfavorable obstetric history (75.2%) compared to non-pregnant women (69.4%) and women with normal pregnancy (69.9%); however, these differences were not significant (Table 4).

HSV-1 IgG antibodies were found in 689 (66.8%, 95% CI = 63.8–69.6%) women. Using ELISA, ten (0.9%, 95% CI = 0.5–1.8%) of the participants were IgM-seropositive, but only one (0.1%, 95% CI = <0.1–0.5%) was confirmed IgM-positive by WB. There was no significant difference in seroprevalence between age groups (63.7–75.0%). The seroprevalence rates were similar in residents of continental (66.9%) and coastal areas (66.6%); however, significant differences were observed among residents of urban and suburban/rural areas (64.7% vs. 71.7%, *p* = 0.030). Obstetric history was not associated with HSV-1 seropositivity (64.8–70.8%) (Table 5).

HSV-2 IgG antibodies were detected in 36 (3.5%, 95% CI = 2.4–4.8%) participants. HSV-2 IgG seropositivity increased significantly with age. None of the participants aged less than 26 years was IgG-seropositive. A progressive increase with age was observed from 1.7% in the 26- to 30-year age group to 9.3% in participants older than 40 years (*p* = 0.005). Area of residence, geographic region, and obstetric history were not associated with HSV-2 IgG seropositivity (Table 6).

### 3.3. Simultaneous TORCH Seroprevalence

Analyzing the simultaneous IgG seroprevalence of two TORCH pathogens, seropositivity to *T. gondii*–RUBV was found in 190 (18.4%; 95% CI = 16.1–20.9%) participants, *T. gondii*–CMV in 146 (14.1%, 95% CI = 12.1–16.4%), and RUBV–CMV in 663 (64.2%; 95% CI = 61.2–67.2%) participants. Only area of residence and geographic region were associated with seropositivity. Residents of suburban/rural areas and those from continental geographic regions were more often seropositive to *T. gondii*–RUBV and *T. gondii*–CMV IgG antibodies, while these differences were not observed for RUBV–CMV seroprevalence (Table 7).

Simultaneous seropositivity to three TORCH pathogens (*T. gondii*–RUBV–CMV) was found in 137 (13.1%; 95% CI = 11.3–15.5%) participants. Suburban/rural area of residence and continental region were also found to be significant risk factors for the simultaneous seropositivity (Table 8).

### 3.4. Risk Analysis for TORCH Seropositivity

Both univariate and multivariate risk analysis showed that area of residence (OR = 1.826, 95% CI = 1.330–2.511, *p* < 0.001; AOR = 1.981, 95% CI = 1.334–2.931, *p* = 0.001) and geographic region (OR = 1.587, 95% CI = 1.120–2.268, *p* = 0.011; AOR = 1.912, 95% CI = 1.217–3.012, *p* = 0.005) were associated with *T. gondii* IgG seropositivity (Table 9). There was no association of age, area of residence, or geographic region with RUBV or CMV IgG seropositivity (Table 10 and Table 11). Suburban/rural area of residence was associated with HSV-1 IgG seropositivity (OR = 1.380, 95% CI = 1.032–1.847, *p* = 0.030; AOR = 1.421, 95% CI = 1.007–2.001, *p* = 0.045) (Table 12). Age was a significant risk factor for HSV-2 IgG seropositivity. The ORs and AORs for a one-year increase in age were 1.164 (95% CI = 1.082–1.251, *p* < 0.001) and 1.130 (95% CI = 1.030–1.240, *p* = 0.010) (Table 13).

## 4. Discussion

The overall TORCH seropositivity in this study (2014–2023) was found to be 20.2% for *T. gondii*, 91.3% for RUBV, 70.5% for CMV, 66.8% for HSV-1, and 3.5% for HSV-2. All detected seroprevalence rates were lower compared to a previous Croatian study (2005–2009) conducted in the same population group (*T. gondii* 29.1%, RUBV 94.6%, CMV 75.3%, HSV-1 78.7% and HSV-2 6.8%) [15]. Better living standards, improved hygiene standards, and safer sexual contact are the possible reasons for declining trends in overall TORCH seroprevalence.

Comparing the overall *T. gondii* seropositivity, the detected seroprevalence rate in Croatia (20.2%) was similar to the seroprevalence in Italy (20.9%, Palermo, 2012–2022) [18], Spain (21%, Albacete, 2006) [19], Poland (22.3%, 2018–2019) [20] and northern Kosovo and Metohija (24.1%, 2011–2012) [21]. Lower seropositivity was found in Serbia (12.7%, Belgrade, 2018–2019) [22], the United Kingdom (9.1%, Kent, 1999–2001; 17.32%, London, 2006–2008) [23,24] and in Turkey (14%, Karabük, 2008) [25], while higher seropositivity was found in France (31.3%, 2016) [26], Germany (39.5%, 2006–2018) [20], and the Netherlands (40.5%, 1995–1996) [27].

Seroepidemiologic studies in Romania showed regional differences in the prevalence of *T. gondii* IgG antibodies, ranging from 38.24% in Bucharest to 55.8% in western Romania [28,29,30,31,32,33,34]. In addition, significant regional differences in seropositivity within France were observed, from 19.1% in Grand Est to 35.1% in Occitanie, while the highest seroprevalence of 50.7% was detected in overseas departments (Guadeloupe, French Guyana, La Réunion, Martinique and Mayotte combined) [26]. Studies from Italy showed similar results. From 2013 to 2017, a seroprevalence study on toxoplasmosis was conducted in childbearing-aged women from Siena (Tuscany, central Italy) and Bari (Apulia, southern Italy) and pregnant women in Bari (2016–2017). The prevalence of seropositive childbearing-aged women in Bari was significantly higher than in Siena (22.4% vs. 12.4%), while a low prevalence (13.8%) was observed among the pregnant women tested [35].

The toxoplasma IgG seroprevalence rates differed regionally in Croatia as well, with significantly higher seropositivity in continental (22.2%) than in coastal regions (15.3%). Eating undercooked pork is one of the main sources of toxoplasmosis [36]. The production and consumption of numerous traditional pork dishes in continental regions may be the explanation for a higher toxoplasma seroprevalence than in coastal regions.

As with the decreasing seroprevalence in Croatia (29.1% seropositive women in 2005–2009 and 20.2% seropositive women in 2014–2023), toxoplasma seropositivity shows declining trends in several countries [32,35,37]. A decreasing trend in the overall seroprevalence of toxoplasmosis from 25–43% to 10–20% was observed in Sweden between 1969 and 1998 in pregnant women born in the Nordic countries who resided in Stockholm [37]. In Romania, seropositivity declined in 10 years from 43.79% (2008–2010) to 38.81% (2015–2018) in pregnant women residing in Timisoara [32].

Numerous studies have shown that toxoplasma seropositivity is age-related, with higher seropositivity as age increases [19,26,38]. In contrast, no significant differences in IgG prevalence between age groups were observed in our study, ranging from 18.8% to 25.6% with a reverse U-shaped seroprevalence curve. Seroprevalence was lowest in the age group of 31 to 35 years. In a 2005–2009 Croatian study, these differences were significant and showed a similar seroprevalence curve with the lowest seroprevalence in the 26- to 35-year group [15].

Our study found significantly higher toxoplasma seropositivity among residents of suburban and rural areas (27.4%) compared to urban areas (17.1%). Significantly higher seroprevalence in rural areas was also observed in a Romanian study (46 vs. 36%) [31], while it was of borderline significance in a Serbian study (22.5 vs. 11.5%) [22]. In Slovakia, women who lived in Bratislava’s surroundings had a considerably higher seropositivity rate (63.5%) than those who lived in Bratislava (36.6%) [39]. In addition to the consumption of pork, more frequent contact with cats, a definite host of *T. gondii*, in rural areas is the probable reason for the differences in the seroprevalence rates, since cat ownership was confirmed to be associated with toxoplasma seroprevalence [25].

A meta-analysis of RUBV prevalence in childbearing-aged and pregnant women that included the period between 2000 and 2016 found a pooled global RUBV seropositivity of 90.7%. When considering subpopulation groups, a seropositivity pooled estimate was 90.6% in pregnant women and 90.5% in childbearing-aged women, with no mention of ongoing pregnancy [40]. In the Croatian childbearing-aged and pregnant women tested in this study, the seroprevalence of RUBV was 91.3%, which is lower than the 94.6% in 2005–2009 [15]. Other European countries showed a seropositivity rate of 93.6% in Ireland [41], 94.4% in Norway [42], 93.4–97.7% in Spain [43,44,45], and 95.8% in Sweden [46]. In the United Kingdom, seroprevalence in Liverpool was lower (93.7%) than average for the northwestern region (96.3%) [47]. Italian studies showed lower seropositivity rates of 85.8% in Messina (2006–2007) [48], 88.6% in Tuscany and 84.3% in Apulia (2014–2016) [49] and 81.2% in Palermo (2012–2022) [18]. Similar seroprevalence of 83.5% was also found in Portugal [50]. Although data on the rubella vaccination status of the participants included in this study were not available, data on measles–mumps–rubella (MMR) vaccine coverage in Croatia from the Reference Center for Epidemiology, Croatian Institute of Public Health showed a decline in the past decade. MMR primo vaccination and revaccination rates in 2013 and 2023 were 93.9/97.1% and 90.1/90.1%, respectively, which impacted the lower seroprevalence rate detected in childbearing-aged women tested in this study.

In Romania, 94.1% of fertile women tested from 2008 to 2010 were rubella IgG-seropositive, while seropositivity was lower (91.5%) in those tested from 2015 to 2018 [51]. A decline in RUBV seropositivity in Croatia is a result of a decrease in immunization coverage, which was also observed in Serbia (92.9% seropositive) [52].

In our study, no significant differences were observed in RUBV seropositivity between age groups (81.3–95.4%), area of residence, geographic region, or obstetric history. Similarly, seroprevalence was stable in different age groups in Romania. In addition, there was no significant difference in seroprevalence among urban and rural populations [51]. In contrast, the highest proportion of seronegative women was found in the youngest age group (15–20 years, 18.7%) compared to 1.9% in the 30- to 35-year age group in northwest England [47]. Similarly, the proportion of RUBV-seronegative women declined with increasing maternal age in Spain [53].

The European studies showed widely differing levels of immunity to CMV in childbearing-aged women. Compared to the seroprevalence of 70.5% observed in our study, very high CMV seropositivity was found in Bosnia and Herzegovina (93%) [54], moderate seropositivity in Italy (70.8%) [55] and Western Europe (73.2%) [56], and lower seroprevalence in the United Kingdom (49%) [57], France (45.6%) [58], Germany (42.3–45.2%) [59,60], and Norway (62.8%) [42]. Studies from Poland showed CMV seroprevalence rates of 76.7% [61] and 62.4%, respectively [62]. Very low seropositivity of 30.4% was found in pregnant Irish women [63].

Similar to decreasing trends in other TORCH pathogens, CMV seroprevalence has also decreased in many European countries. A cross-sectional study conducted in Finland at three time points (1992, 2002, and 2012) showed that seroprevalence decreased significantly from 84.5% to 71.5% over 20 years [64]. Our results also showed a decline in CMV IgG seropositivity. In a previous Croatian study (2005–2009), 78.7% of childbearing-aged women were CMV-seropositive [15] compared to 70.5% in this study. Romania found stable seropositivity rates of 93.68% and 94.96% in 2013–2016 and 2019–2022, respectively, in southwestern regions [65] and a decrease from 94.6% (2008–2010) to 91.80% (2015–2018) in the western region [66].

In our study, no significant differences in CMV IgG seropositivity were observed among age groups, although seroprevalence rates were lower in groups aged up to 25 years (62.5% and 66.7%, respectively) compared to groups aged above 26 years (69.7–72.4%). Stable but higher seroprevalence rates were also found in Croatia in 2005–2009 [15]. Similarly, no significant differences in seropositivity by age group were found in Italy [55]. In addition, no correlation between seroprevalence and maternal age was observed in one Polish study [61]. However, in another study from Poland, seroprevalence differed significantly between age-stratified groups, with the highest IgG prevalence in women above 36 years of age (76.2%) compared to 58.5–66.0% in younger women [62]. Moreover, in the United Kingdom, a significant increase in CMV seroprevalence was observed with maternal age from 50.9% to 75.5% [57].

In contrast to Romanian studies, which found higher CMV seropositivity in pregnant women residing in rural areas [65,66], our study found no difference in seroprevalence between residents of suburban/rural and urban areas (73.9% vs. 69.1%).

Like other TORCH pathogens, the reported HSV seroprevalence rates were heterogeneous within Europe. In our study, HSV-1 and HSV-2 seroprevalence was 66.8% and 3.5%, respectively. HSV-1 seropositivity was found to be 79.4% in Switzerland [67], 88% in Estonia [68], 88.2% in France [69], 91.2% in Italy [70], and 94.7% in Turkey [71]. In Finland, HSV-1 seropositivity varied in different studies, ranging from 46.8% [72] to 69.5% [64]. HSV-2 seroprevalence was high in Estonia (24%) [68], Switzerland (21.2%) [67], and lower in Italy (9.9%) [70] and Turkey (8.2%) [71]. Both HSV-1 and HSV-2 seroprevalence differed between regions in the Netherlands due to the multiethnic composition of the population. Seroprevalence rates were 61% and 11% in Nijmegen, 73% and 35% in Amsterdam, and 75% and 27% in Rotterdam [73].

Our study found no difference in either HSV-1 or HSV-2 IgG seroprevalence among residents of continental and coastal regions. However, higher HSV-1 seropositivity was observed in women from suburban/rural areas (71.7%) than women from urban areas (64.7%). The higher HSV-1 seroprevalence in rural areas could be attributed to the lifestyle of the rural population, typically living in large households with many children and relatives. Since primary infections mainly occur in preschool- and school-aged children, close contact with these cohorts is associated with an increased risk of HSV-1 transmission [2].

Our study showed a significant increase in HSV-2 seropositivity with age. All women aged less than 25 years were HSV-2 IgG-seronegative, while a progressive increase in seroprevalence was observed starting with the 26- to 30-year age group, from 1.7 to 9.3%. Higher seropositivity in older groups reflects longer and cumulative exposure to the virus. Similar to our results, an increase in HSV-2 seropositivity with age was observed in many studies [67,72,74]. No association of HSV-2 seroprevalence with age was found in Romania, with peak seroprevalence (18.3%) between 30 and 34 years of age and a slight decrease thereafter [75].

While obstetric history was not associated with HSV-1 or HSV-2 seroprevalence in Croatian childbearing-aged or pregnant women, a history of abortion was associated with HSV-2 seropositivity in German and Hungarian studies [76,77].

A Romanian study analyzed the simultaneous seroprevalence of TORCH pathogens in childbearing-aged women. Similar to each TORCH pathogen, the proportion of women simultaneously IgG-seropositive decreased over time: *T. gondii*–CMV 41.4% vs. 36.1%, *T. gondii*–RUBV 41.8% vs. 35.7%, CMV–RUBV 88.9% vs. 83.6%, and *T. gondii–*CMV–RUBV 39.6% vs. 33.2% [78]. Simultaneous seropositivity to two or three TORCH pathogens in participants included in our study was generally low (*T. gondii*–RUBV 18.4%, *T. gondii*–CMV 14.1%, *T. gondii*–RUBV–CMV 13.1%) and moderate for RUBV–CMV (64.2%). In a previous Croatian study, simultaneous seropositivity was not analyzed; therefore, it was not possible to compare seroprevalence trends over time. When comparing women from urban and suburban–rural areas, simultaneous seroprevalence was higher in suburban and rural regions, which is in line with the results from Romania [78].

One recently published study analyzed the impact of latent CMV infections on spontaneous abortion history and pregnancy outcomes. In healthy women, latent CMV infection does not affect the risk of complications, while borderline-significant higher prevalence of miscarriage history was observed in women with latent CMV infection [79]. Furthermore, the observed differences between the rate of pregnancy complications in groups of pregnant women with and without latent *T. gondii* infection were not significant [30]. In our study, data on pregnancy complications and outcomes were not available, which is one of the limitations of the study.

In addition, a limitation of this study that needs to be addressed is the small number of participants in the youngest (≤25 years) and oldest (>40 years) age groups, which should be considered when interpreting the results.

## 5. Conclusions

Based on the decline in seroprevalence rates observed in many European countries, a similar trend was expected in Croatia. The results of this study confirmed this hypothesis, showing a decrease in TORCH-seroprevalence rates in Croatian childbearing-aged women in 2014–2023 compared to 2005–2009.

Information on TORCH serostatus in childbearing-aged women is important to define seronegative women who are at risk of primary infections during pregnancy. Although cases of rubella reinfections in previously vaccinated seropositive women during pregnancy are reported in the literature, CRS is rarely recorded [80,81]. In addition, CMV reactivation or reinfection during pregnancy can lead to transient viremia and fetal infection, but such infections tend to be less severe and newborns are usually asymptomatic [82,83]. Congenital toxoplasmosis as a result of reinfection in immunocompetent pregnant women or reactivation in pregnant women with altered immune status is exceptional [84]. Since rubella is a vaccine-preventable disease, serological testing of childbearing-aged women is encouraged with the aim of vaccinating seronegative individuals before pregnancy.

The results of our study also impact other population groups, especially the immunocompromised, who are at risk of toxoplasmosis, HSV, and CMV infection. Similarly to the observed declining seroprevalence in childbearing-aged women, decreasing seroprevalence and increased susceptibility to infections probably occur in this population, which should be kept in mind. Therefore, the present results also highlight the need to monitor these pathogens in other high-risk population groups as well.

## Figures and Tables

**Table 1:** Serology methods used for detection of TORCH antibodies.

TORCH Pathogen	Method (Reference Values)			
	IgM Antibodies	IgG Antibodies	IgG Avidity	Western Blot
*T. gondii*	ELFA (Index < 0.55 negative, 0.55–0.65 borderline, >0.65 positive)	ELFA (IU/mL < 4 negative, 4–8 borderline, >8 positive)	ELFA (Index < 0.3 low, 0.3–0.5 borderline, >0.5 high)	
RUBV	ELISA (NTU < 9 negative, 9–11 borderline, >11 positive)	ELISA (IU/mL < 10 negative, 10–15 borderline, 15 positive)	ELISA (AI % < 40 low, 40–60 borderline, >60% high)	
CMV	ELISA (AI < 9 negative, 9–11 borderline, >11 positive)	ELISA (AI < 9 negative, 9–11 borderline, >11 positive)	ELISA (AI % < 40 low, 40–60 borderline, >60% high)	
HSV-1	ELISA (VE < 9 negative, 9–11 borderline, >11 positive)	ELISA (VE < 9 negative, 9–11 borderline, >11 positive)		Positive, borderline, negative
HSV-2	ELISA (VE < 9 negative, 9–11 borderline, >11 positive)	ELISA (VE < 9 negative, 9–11 borderline, >11 positive)		Positive, borderline, negative

ELFA = enzyme-linked fluorescence assay, ELISA = enzyme-linked immunosorbent assay; NTU = Novatec units, AI = avidity index, IU/mL = international units/mL, VE = Virotech units.

**Table 2:** Prevalence of *Toxoplasma gondii* antibodies.

Characteristic		Tested N (%)	*Toxoplasma gondii* IgM			*Toxoplasma gondii* IgG		
			N (%)	95% CI	*p*	N (%)	95% CI	*p*
Age group	≤20 years	16 (1.6)	1 (6.3)	0.1–30.2	0.007	4 (25.0)	7.3–52.4	0.896
	21–25 years	87 (8.4)	0 (0)	NA		18 (20.7)	12.7–30.7	
	26–30 years	300 (29.1)	1 (0.3)	<0.1–1.2		63 (21.0)	16.5–26.1	
	31–35 years	373 (36.1)	2 (0.5)	0.1–1.9		70 (18.8)	14.9–23.1	
	36–40 years	213 (20.6)	0 (0)	NA		42 (19.7)	14.6–25.7	
	>40 years	43 (4.2)	0 (0)	NA		11 (25.6)	13.5–41.2	
Area of residence	Urban	725 (70.3)	2 (0.3)	<0.1–1.0	0.375	124 (17.1)	14.4–20	<0.001
	Suburban/rural	307 (29.7)	2 (0.7)	0.1–2.3		84 (27.4)	22.5–32.7	
Geographic region	Continental	724 (70.2)	3 (0.4)	<0.1–1.2	0.832	161 (22.2)	19.3–25.4	0.011
	Coastal	308 (29.8)	1 (0.3)	<0.1–1.8		47 (15.3)	11.4–19.8	
Obstetric history	Non-pregnant	271 (26.3)	0 (0)	NA	0.247	64 (23.6)	18.7–29.1	0.204
	Normal pregnancy	608 (58.9)	4 0.7)	0.2–1.7		118 (19.4)	16.3–22.8	
	Unfavorable obstetric history	153 (14.8)	0 (0)	NA		26 (17.0)	11.4–23.9	

NA = not applicable, CI = confidence interval.

**Table 3:** Prevalence of rubella virus antibodies.

Characteristic		Tested N (%)	Rubella Virus IgM			Rubella Virus IgG		
			N (%)	95% CI	*p*	N (%)	95% CI	*p*
Age group	≤20 years	16 (1.6)	0 (0)	NA	0.378	13 (81.3)	54.4–96.0	0.319
	21–25 years	87 (8.4)	1 (1.1)	<0.1–6.2		83 (95.4)	88.6–98.7	
	26–30 years	300 (29.1)	0 (0)	NA		268 (89.3)	85.3–92.4	
	31–35 years	373 (36.1)	1 (0.3)	<0.1–1.5		341 (91.4)	88.1–94.1	
	36–40 years	213 (20.6)	0 (0)	NA		197 (92.5)	88.1–95.6	
	>40 years	43 (4.2)	0 (0)	NA		40 (93.0)	80.9–98.5	
Area of residence	Urban	725 (70.3)	2 (0.3)	<0.1–1	0.357	662 (91.3)	89–93.3	0.956
	Suburban/rural	307 (29.7)	0 (0)	NA		280 (91.2)	87.5–94.1	
Geographic region	Continental	724 (70.2)	2 (0.3)	<0.1–1	0.356	664 (91.7)	89.5–93.6	0.449
	Coastal	308 (29.8)	0 (0)	NA		278 (90.3)	86.4–93.3	
Obstetric history	Non-pregnant	271 (26.3)	0 (0)	NA	0.498	248 (91.5)	87.5–94.5	0.976
	Normal pregnancy	608 (58.9)	2 (0.3)	<0.1–1.2		554 (91.1)	88.6–93.3	
	Unfavorable obstetric history	153 (14.8)	0 (0)	NA		140 (90.5)	85.9–95.4	

NA = not applicable, CI = confidence interval.

**Table 4:** Prevalence of cytomegalovirus antibodies.

Characteristic		Tested N (%)	Cytomegalovirus IgM			Cytomegalovirus IgG		
			N (%)	95% CI	*p*	N (%)	95% CI	*p*
Age group	≤20 years	16 (1.6)	1 (6.3)	0.2–39.2	0.248	10 (62.5)	35.4–84.8	0.863
	21–25 years	87 (8.4)	10 (11.5)	5.7–20.1		58 (66.7)	55.7–76.4	
	26–30 years	300 (29.1)	22 (7.3)	4.7–10.9		206 (69.7)	63.1–73.9	
	31–35 years	373 (36.1)	17 (4.6)	2.7–7.2		270 (72.4)	67.5–76.9	
	36–40 years	213 (20.6)	13 (6.1)	3.3–10.2		150 (70.4)	63.8–76.5	
	>40 years	43 (4.2)	2 (4.7)	0.6–15.8		31 (72.1)	56.3–84.7	
Area of residence	Urban	725 (70.3)	47 (6.5)	4.8–8.5	0.708	501 (69.1)	65.6–72.5	0.119
	Suburban/rural	307 (29.7)	18 (5.9)	3.5–9.1		227 (73.9)	68.7–78.8	
Geographic region	Continental	724 (70.2	41 (5.7)	4.1–7.6	0.198	507 (70.0)	66.5–73.3	0.587
	Coastal	308 (29.8	24 (7.8)	5.1–11.4		221 (71.8)	66.4–76.7	
Obstetric history	Non-pregnant	271 (26.3)	21 (7.7)	4.9–11.6	0.495	188 (69.4)	63.5–74.8	0.393
	Normal pregnancy	608 (58.9)	36 (5.9)	4.2–8.1		425 (69.9)	66.1–73.5	
	Unfavorable obstetric history	153 (14.8)	8 (5.2)	2.3–10		115 (75.2)	67.5–81.8	

CI = confidence interval.

**Table 5:** Prevalence of herpes simplex virus type 1 antibodies.

Characteristic		Tested N (%)	Herpes Simplex Type 1 IgM			Herpes Simplex Type 1 IgG		
			N (%)	95% CI	*p*	N (%)	95% CI	*p*
Age group	≤20 years	16 (1.6)	0 (0)	NA	0.538	12 (75.0)	47.6–92.7	0.598
	21–25 years	87 (8.4)	0 (0)	NA		57 (65.5)	54.6–75.4	
	26–30 years	300 (29.1)	5 (1.7)	0.5–3.8		191 (63.7)	57.9–69.1	
	31–35 years	373 (36.1)	2 (0.5)	<0.1–1.9		249 (66.8)	61.7–71.5	
	36–40 years	213 (20.6)	2 (0.9)	0.1–3.4		148 (69.5)	62.8–75.6	
	>40 years	43 (4.2)	1 (2.3)	<0.1–12.3		32 (74.4)	58.8–86.5	
Area of residence	Urban	725 (70.3)	9 (1.2)	0.6–2.3	0.170	469 (64.7)	61.1–68.2	0.030
	Suburban/rural	307 (29.7)	1 (0.3)	<0.1–1.8		220 (71.7)	66.3–76.6	
Geographic region	Continental	724 (70.2)	7 (1.0)	0.4–2	0.991	484 (66.9)	63.3–70.3	0.927
	Coastal	308 (29.8)	3 (1.0)	0.2–2.8		205 (66.6)	61–71.8	
Obstetric history	Non-pregnant	271 (26.3)	2 (0.7)	<0.1–2.6	0.300	192 (70.8)	65–76.2	0.211
	Normal pregnancy	608 (58.9)	8 (1.3)	0.6–2.6		394 (64.8)	60.9–68.6	
	Unfavorable obstetric history	153 (14.8)	0 (0)	NA		103 (67.3)	59.3–74.7	

NA = not applicable, CI = confidence interval.

**Table 6:** Prevalence of herpes simplex virus type 2 antibodies.

Characteristic		Tested N (%)	Herpes Simplex Type 2 IgM			Herpes Simplex Type 2 IgG		
			N (%)	95% CI	*p*	N (%)	95% CI	*p*
Age group	≤20 years	16 (1.6)	0 (0)	NA	0.880	0 (0)	NA	0.005
	21–25 years	87 (8.4)	0 (0)	NA		0 (0)	NA	
	26–30 years	300 (29.1)	0 (0)	NA		5 (1.7)	0.5–3.8	
	31–35 years	373 (36.1)	1 (0.3)	<0.1–1.5		13 (3.5)	1.9–5.9	
	36–40 years	213 (20.6)	0 (0)	NA		14 (6.6)	3.6–10.8	
	>40 years	43 (4.2)	0 (0)	NA		4 (9.3)	2.6–22.1	
Area of residence	Urban	725 (70.3)	0 (0)	NA	0.124	27 (3.7)	2.5–5.4	0.526
	Suburban/rural	307 (29.7)	1 (0.3)	<0.1–1.8		9 (2.9)	1.3–5.5	
Geographic region	Continental	724 (70.2)	1 (0.1)	<0.1–0.8	0.514	27 (3.7)	2.5–5.4	0.518
	Coastal	308 (29.8)	0 (0)	NA		9 (2.9)	1.3–5.5	
Obstetric history	Non-pregnant	271 (26.3)	0 (0)	NA	0.706	14 (5.2)	2.9–8.5	0.106
	Normal pregnancy	608 (58.9)	1 (0.2)	<0.1–1		20 (3.3)	2–5	
	Unfavorable obstetric history	153 (14.8)	0 (0)	NA		2 (1.3)	0.2–4.6	

NA = not applicable, CI = confidence interval.

**Table 7:** Simultaneous IgG seroprevalence of two TORCH pathogens.

Characteristic	Tested N (%)	*Toxoplasma gondii*– Rubella Virus IgG			*Toxoplasma gondii*– Cytomegalovirus IgG			Rubella Virus– Cytomegalovirus IgG		
		N (%)	95% CI	*p*	N (%)	95% CI	*p*	N (%)	95% CI	*p*
Age group										
≤20 years	16 (1.6)	4 (25.0)	7.0–52.4	0.754	2 (12.5)	1.5–38.3	0.962	7 (43.8)	19.8–79.1	0.408
21–25 years	87 (8.4)	18 (20.7)	12.7–30.7		12 (13.8)	7.3–22.9		56 (64.4)	53.4–74.4	
26–30 years	300 (29.1)	59 (19.7)	15.3–24.6		44 (14.7)	10.9–19.2		185 (61.7)	55.9–67.2	
31–35 years	373 (36.1)	63 (16.9)	13.2–21.1		52 (13.9)	10.6–17.9		250 (67.0)	62.0–71.8	
36–40 years	213 (20.6)	36 (16.9)	12.1–22.6		28 (13.1)	8.9–18.4		137 (64.3)	57.5–70.7	
>40 years	43 (4.2)	10 (23.3)	11.8–38.6		8 (18.6)	8.4–33.4		28 (65.1)	49.1–79.0	
Area of residence										
Urban	725 (70.3)	112 (15.4)	12.9–18.3	<0.001	85 (11.7)	9.5–14.3	<0.001	458 (63.2)	59.5–66.7	0.270
Suburban/rural	307 (29.7)	78 (25.4)	20.6–30.7		61 (19.9)	15.6–24.5		205 (66.8)	61.2–72.0	
Geographic region										
Continental	724 (70.2)	145 (20.0)	17.2–23.1	0.04	115 (15.9)	13.3–18.8	0.014	465 (64.2)	60.6–67.7	0.986
Coastal	308 (29.8)	45 (14.0)	10.9–19.1		31 (10.1)	6.9–14		198 (63.3)	58.7–69.6	
Obstetric history										
Non-pregnant	271 (26.3)	57 (21.0)	16.3–26.4	0.351	44 (16.2)	12.1–21.2		172 (63.5)	57.4–69.2	0.281
Normal pregnancy	608 (58.9)	109 (17.9)	15–21.2		83 (13.7)	11.0–16.6		384 (63.2)	59.2–67.0	
Unfavorable obstetric history	153 (14.8)	24 (15.7)	10.3–22.4		19 (12.4)	7.6–18.7		107 (69.4)	62.0–77.1	

CI = confidence interval.

**Table 8:** Simultaneous IgG seroprevalence of three TORCH pathogens.

Characteristic		Tested N (%)	*Toxoplasma gondii*–Rubella Virus–Cytomegalovirus IgG		
			N (%)	95% CI	*p*
Age group	≤20 years	16 (1.6)	2 (12.5)	1.6–38.3	0.946
	21–25 years	87 (8.4)	12 (13.8)	7.3–22.9	
	26–30 years	300 (29.1)	42 (14.0)	10.3–18.4	
	31–35 years	373 (36.1)	49 (13.1)	9.9–17.0	
	36–40 years	213 (20.6)	25 (11.7)	7.7–16.8	
	>40 years	43 (4.2)	7 (16.3)	6.8–30.7	
Area of residence	Urban	725 (70.3)	80 (11.0)	8.8–13.5	0.002
	Suburban/rural	307 (29.7)	57 (18.6)	14.4–23.4	
Geographic region	Continental	724 (70.2)	107 (14.8)	12.3–17.6	0.029
	Coastal	308 (29.8)	30 (9.7)	6.7–13.6	
	Non-pregnant	271 (26.3)	40 (14.8)	10.8–19.6	0.697
Obstetric history	Normal pregnancy	608 (58.9)	78 (12.8)	10.3–15.8	
	Unfavorable obstetric history	153 (14.8)	19 (12.4)	7.6–18.7	

CI = confidence interval.

**Table 9:** Univariate and multivariate risk for *Toxoplasma gondii* IgG seropositivity.

Characteristic	OR	95% CI	*p*	AOR	95% CI	*p*
Age (one-year increase)	0.990	0.960–1.030	0.614	1.000	0.961–1.040	0.854
Suburban/rural (Ref.) vs. urban area of residence	1.826	1.330–2.511	<0.001	1.981	1.334–2.931	0.001
Continental (Ref.) vs. coastal geographic region	1.587	1.120–2.268	0.011	1.912	1.217–3.012	0.005
Unfavorable obstetric history (Ref.) vs. normal pregnancy	0.850	0.533–1.357	0.496	0.882	0.547–1.421	0.605

OR = odds ratio, AOR = adjusted odds ratio, CI = confidence interval.

**Table 10:** Univariate and multivariate risk for rubella virus IgG seropositivity.

Characteristic	OR	95% CI	*p*	AOR	95% CI	*p*
Age (one-year increase)	1.016	0.975–1.059	0.473	1.014	0.964–1.068	0.585
Suburban/rural (Ref.) vs. urban area of residence	0.986	0.616–1.582	0.958	1.023	0.585–1.788	0.937
Continental (Ref.) vs. coastal geographic region	1.195	0.754–1.912	0.450	1.458	0.855–2.488	0.167
Unfavorable obstetric history (Ref.) vs. normal pregnancy	1.050	0.557–1.977	0.881	1.097	0.579–2.079	0.772

OR = odds ratio, AOR = adjusted odds ratio, CI = confidence interval.

**Table 11:** Univariate and multivariate risk for cytomegalovirus IgG seropositivity.

Characteristic	OR	95% CI	*p*	AOR	95% CI	*p*
Age (one-year increase)	1.001	0.982–1.035	0.558	1.002	0.970–1.034	0.912
Suburban/rural (Ref.) vs. urban area of residence	1.269	0.940–1.712	0.120	1.289	0.899–1.849	0.168
Continental (Ref.) vs. coastal geographic region	0.920	0.684–1.235	0.578	0.983	0.690–1.403	0.927
Unfavorable obstetric history (Ref.) vs. normal pregnancy	1.303	0.869–1.955	0.201	1.288	0.855–1.939	0.226

OR = odds ratio, AOR = adjusted odds ratio, CI = confidence interval.

**Table 12:** Univariate and multivariate risk for herpes simplex virus type 1 IgG seropositivity.

Characteristic	OR	95% CI	*p*	AOR	95% CI	*p*
Age (one-year increase)	1.016	0.990–1.042	0.231	1.009	0.978–1.040	0.588
Suburban/rural (Ref.) vs. urban area of residence	1.380	1.032–1.847	0.030	1.421	1.007–2.001	0.045
Continental (Ref.) vs. coastal geographic region	1.013	0.764–1.344	0.927	1.035	0.740–1.449	0.841
Unfavorable obstetric history (Ref.) vs. normal pregnancy	1.119	0.768–1.631	0.559	1.106	0.756–1.619	0.604

OR = odds ratio, AOR = adjusted odds ratio, CI = confidence interval.

**Table 13:** Univariate and multivariate risk for herpes simplex virus type 2 IgG seropositivity.

Characteristic	OR	95% CI	*p*	AOR	95% CI	*p*
Age (one-year increase)	1.164	1.082–1.251	<0.001	1.130	1.030–1.240	0.010
Suburban/rural (Ref.) vs. urban area of residence	0.781	0.363–1.680	0.527	0.939	0.333–1.651	0.906
Continental (Ref.) vs. coastal geographic region	1.287	0.598–2.771	0.519	1.695	0.553–5.181	0.356
Unfavorable obstetric history (Ref.) vs. normal pregnancy	0.389	0.900–1.684	0.207	0.394	0.900–1.724	0.216

OR = odds ratio, AOR = adjusted odds ratio, CI = confidence interval.

## Data Availability

Data are contained within the article.
